# The Vietnamese version of the Perceived Stress Scale (PSS-10): Translation equivalence and psychometric properties among older women

**DOI:** 10.1186/s12888-017-1221-6

**Published:** 2017-02-06

**Authors:** Tiet-Hanh Dao-Tran, Debra Anderson, Charrlotte Seib

**Affiliations:** 1 Faculty of Nursing and Medical Technology, University of Medicine and Pharmacy, HCMC, Vietnam; 2 Nursing School, Queensland University of Technology, N602, N block, Kelvin Grove campus of QUT, Victoria Park Rd, Kelvin Grove, Brisbane, QLD Australia QLD4059; 3 Head of School of Nursing and Midwifery, Griffith University, Brisbane, QLD Australia; 4 School of Nursing and Midwifery, Griffith University, Brisbane, QLD Australia; 5 Nursing School, Queensland University of Technology, Brisbane, QLD Australia QLD4059

**Keywords:** Perceived Stress Scale, Older women, Reliability, Validity, Vietnamese

## Abstract

**Background:**

The Perceived Stress Scale 10 item (PSS-10) has been translated into more than 20 languages and used widely in different populations. Yet, to date, no study has tested psychometric properties of the instrument among older women and there is no Vietnamese version of the instrument.

**Methods:**

This study translated the PSS-10 into Vietnamese and assessed Vietnamese version of the Perceived Stress Scale 10 items (V-PSS-10) for translation equivalence, face validity, construct validity, correlations, internal consistency reliability, and test-retest reliability among 473 women aged 60 and over.

**Results:**

The study found that V-PSS-10 retained the original meaning and was understood by Vietnamese older women. An exploratory factor analysis of the V-PSS-10 yielded a two-factor structure, and these two factors were significantly correlated (0.56, p < .01) with all item loadings exceeded .50. The V-PSS-10 score was positively correlated with general sleep disturbance (ρ = .12, *p* < .05), CES-D score for depression symptoms (ρ = .60, *p* < .01), and negatively correlated with mental (ρ = −.46, *p* < .01), and physical health scores (ρ = −.19, *p* < .01). The Cronbach’s alpha for the V-PSS-10 was .80, and the test-retest correlation at one month’s interval was .43.

**Conclusion:**

Findings from this study suggest that the V-PSS-10 has acceptable validity and reliability levels among older women. The V-PSS-10 can be used to measure perceived stress in future research and practice. However, future research would be useful to further endorse the validity and reliability of the V-PSS-10.

## Background

Stress has been linked with a number of health issues including hypertension [1], coronary heart disease [2], stroke [3], cancer [4], diabetes [5], anxiety [6], and depression [7]. Because of genetic, biological factors and sex hormones, women are more likely to experience stress than men [8, 9]. This difference has been reported among women, who are older, widowed, have a disability, are members of a minority ethnic group, poorly educated, and living in undesirable conditions and poverty [8, 9].

Concerning stress measurement, Cohen [10] has suggested that measuring stress as an appraisal of events rather than measuring the events themselves is likely to be sensitive, reliable and less limited to a specific group of participants or contexts. So, stress appraisal instruments have been widely used in a variety of research settings [2, 11, 12]. One popular stress appraisal instrument is the Perceived Stress Scale 10 items (PSS-10) [13]. The Perceived Stress Scale (PSS) has been validated and used in a variety of population groups and translated into over 20 languages with internal consistency estimates using Cronbach’s alpha ranging from .67 to .91 [14–16], and test-retest- reliability ranging from .53 to .83 [14–16]. The PSS-10 has been found to provide better predictions for psychological symptoms, physical symptoms and utilisation of health services than other similar instruments [14, 17]. However, to date, no study has tested psychometric properties of the instrument among older women.

Traditionally, Vietnamese women were born with the thought of “one boy can be counted but not ten girls” [18]. Consequently, girls do not generally receive equivalent education and vocational training to boys, and therefore have fewer opportunities for paid employment [18]. Moreover, in many instances, girls are not able to choose their marital partner [19]. In addition, during the last five decades, Vietnam has gone through several dramatic changes. These have included: the wars; the mass flow of refugees; the most serious ever economic crisis, and dramatically socio-economic increase [20]. These events may cause women in Vietnam to experience stress and be increasingly vulnerable to deteriorating health, especially mental disorders [21]. Yet, our knowledge about their stress is still limited due to the lack of a valid measurement for use in Vietnam. This study translated the PSS-10 into Vietnamese, examined translation equivalence, construct validity, correlations, internal consistency and test-retest reliability of the V-PSS-10 in a sample of older women. It is hoped that through the development of a valid and reliable V-PSS-10, research opportunities will be created that will enhance our understanding of stress perception in Vietnamese, an understudied population.

## Methods

### Translation and evaluation

The translation and back-translation process developed by Sousa and Rojjanasrirat [22] was used to develop a Vietnamese version of the Perceived Stress Scale. In this process, two independent bilingual translators translated the English version into Vietnamese. Then, a consensus meeting among the two translators and the bilingual researcher was arranged to achieve agreement on the final Vietnamese version. Next, the Vietnamese version was sent to three bilingual experienced researchers in the field of population health and women’s health to examine the translation equivalence between the Vietnamese version and the English version. These experts gave their opinion on whether the language used in the Vietnamese version was accurate and clear for older women in Vietnam. Each of the three experts independently rated the language used in the translation on a 5-point Likert scale (one = strongly disagree, five = strongly agree). For evaluating translation equivalence of the instrument, the final proportion of the experts’ agreement on the translation was used. After that, the Vietnamese version was back translated into English by another two independent translators, and again consensus and agreement was achieved on the English back-translation version. Finally, the back-translation version was compared with the original English version by two independent native English speaking experts. These experts also gave their opinion on whether the backed translated version of the instrument kept the same meaning as the original English version for each item on a five-point Likert scale (one = strongly disagree, five = strongly agree).

### Psychometric evaluation

When the translation and back-translation procedure was completed, exploratory factor analysis (EFA) was performed for determining the number of factors in the V-PSS-10’s structure. This study further investigated the correlations between V-PSS-10, and sleep disturbance, depression, mental health and physical health. Finally, the V-PSS-10 was examined for the internal consistency and test-retest reliability at one month.

#### Participants

The study involved 473 older women in Vietnam. Eligibility criteria included: (1) Vietnamese women; (2) 60 years old and above; (3) able to communicate in Vietnamese, and; (4) able to give informed written consent. The potential participants were recruited through Elderly Unions, which are organisations that manage and organise activities for people aged 60 and over in the community. Stratified random sampling was performed on the administrative management lists (provided by the Elders Unions in each locale) to collect data from 16 rural and urban suburbs in Vietnam.

#### Procedure

The researcher contacted the Elderly Unions and was provided with a list of women aged 60 and over in their catchment. With the support from the Unions, potential participants were contacted and invited to participate in the study. Informed written consent was gained prior to data collection. Due to the variation of the participants’ education and vison capacity, individual interviews using a structured questionnaire were used to collect data. Participants provided answers in a private space at either their residences or a community office.

#### Instruments

The Perceived Stress Scale (PSS) [13] was developed to appraise whether participants considered their life to be unpredictable, uncontrollable, or overloaded. Originally, the instrument had 14 self-report items [13] and included both negatively and positively worded items, but it has more recently been shortened to 10 items and 4 items without loss of fidelity [14]. In each question, the respondent is asked how often they felt a certain way and how often they have experienced particular thoughts and feelings during the last month. Responses are rated from 0 (never) to 4 (very often). The PSS-10 [17] is summed to provide possible scores between 0 and 40. Higher scores represent an increased likelihood that environmental demands exceed an individual’s ability to cope. The scale was originally validated in 2,387 participants in the United States with a reported Cronbach’s alpha of .78. It has been validated and used in a variety of population groups and translated into over 20 languages [10]. Cronbach’s alphas have ranged from .67 to .91 [14], and test-retest- reliabilities of > .70 [14].

The General Sleep Disturbance Scale (GSDS) [23] was used to measure sleeping disturbance. The GSDS consists of 21 self-report items of sleep disturbances. Questions pertain to a variety of general sleep issues, including: problems initiating sleep, waking up during sleep, waking too early from sleep, quality of sleep, quantity of sleep, fatigue and alertness at work, and the use of substances to induce sleep. In each question, the respondent is asked how many days in the last 7 days they have experienced the sleep problem. The answers are rated from 0 (never) to seven (everyday). Total possible scores for the GSDS ranged from 0 to 147. Total scores of 43 or above represent general sleep disturbance [23]. The GSDS has a Cronbach’s alpha of .88. This instrument has also been translated into Vietnamese with a reported Cronbach’s alpha of .81.

The Centre of Epidemiologic Studies Depression Scale (CES-D Scale) [24] was used to measure depression. The CES-D Scale consists of 20 self-report items to describe how many days in the last 7 days a person feels or behaves in a certain way [24]. The answers are rated from 0 (less than 1 day) to 3 (5–7 days) [24]. The possible scores range from zero to 60, with the higher scores indicating the presence of more symptoms. Scores between 16 and 26 suggest mild depression, and scores ≥ 27 suggest major depression [25]. The CES-D Scale has demonstrated good moderate test- retest reliability and good criterion related validity [24]. The Cronbach’s alpha for this instrument ranged from .85 to.90 [24]. Test –retest reliability for the CES-D Scale determined in 2-week, 4-week, 6-week, and 8-week intervals apart were .51, .67, .59, and .59 respectively [24]. This instrument has also been translated into Vietnamese and had a Cronbach’s alpha of .90.

The Short-Form 12 items (SF-12) [26] were used to measure general physical and mental health. The SF-12 is a self-report questionnaire, including 12 questions for eight dimensions of health: general health (1 items), mental health (2 items), physical functioning (2 items), bodily pain (1 item), role limitation related to physical health problems (2 items), role limitation due to emotional health problems (2 items), vitality (1 item) and social functioning (1 items) [26]. The SF-12 was scored using a standard scoring procedure and this allowed the score for the whole scale to range from 0 to 100. The reported Cronbach’s alpha for this instrument was .79 [26]. The SF-12 has also demonstrated adequate test-retest reliability with Physical health component scores (PCS) = .89; Mental health component scores (MCS) = .76 [26]. This instrument has also been translated into Vietnamese with a Cronbach’s alpha of .88.

#### Statistical data analysis

Data analysis was performed using the Statistical Package for Social Sciences SPSS 22.0 [27]. Descriptive analysis was used to describe the continuous variables; frequency and percentage were used for categorical variables [28].

To assess construct validity of V-PSS-10, exploratory factor analysis (EFA) was performed on data from 473 Vietnamese older women for determining the number of factors in PSS-10’s structure. It is recommended that the number of items and number of cases ratio should be greater than 1:10 [29, 30], so this sample size (N = 473) was adequate for EFA. The sample adequacy was also assessed by the Kaiser-Mayer-Olkin (KMO) measure of sampling adequacy. Initial determination of the appropriate number of factors to retain was assessed with the Kaiser criterion of eigenvalues [31] and a scree plot [32]. For the eigenvalue criterion, factors with eigenvalues larger than 1 were kept [31]. For the Scree test, the number of factors is determined by locating the last substantial leap in the magnitude of the eigenvalues in the plot [32]. Items with a loading of .40 and above were accepted [30, 33]. Principal Axing Factoring extraction [15] with Promax rotation methods [34] were applied in EFA. Based on a previous study finding, it was expected that V-PSS-10 would have two factors [14]. Then, simple regression data analysis was used to explore the correlation between V-PSS-10 and sleep disturbance, depression, and health-related quality of life. Significance was set at α = .05 [28]. The internal consistency values were assessed using Cronbach’s alpha [35]. It was determined that if the Cronbach’s alpha reached 0.7 or above [36], the tool would be deemed to have internal consistency. Further test-retest reliability over one month was assessed using a Spearman Rho correlation as the data were not normally distributed [28].

## Results

### Translation equivalence and face validity

All three bilingual Vietnamese-English experts’ independent ratings indicated that the V- PSS-10 kept the meaning of the original English version and the language used in V-PSS-10 was clear for older women in Vietnam to understand. To be specific, two out of the three bilingual English-Vietnamese experts strongly agreed and one agreed that all items kept the same meaning of the original English version and were easily understood by Vietnamese older women. Comparing the original English version and the back-translated English version of the instrument, two native English experts agreed strongly that the back- translated version retained equivalent meaning of the original English version for all items.

### Descriptive statistics

Psychometric properties were examined on the data of 473 Vietnamese older women. Overall, the participants were aged 60 –s 94 years (median = 68.0). The majority of the sample lived in rural areas (66%, *n* = 312), believed in Buddhism (93%, *n* = 438), had completed primary school or less (75.2%, *n* = 354), and were not currently employed (70.2%, *n* = 332). Around half of the women lived with their partners (51.2%, *n* = 241), and had low income (53.5%, *n* = 252).

### Construct validity, and correlates of perceived stress

**Fig. 1:**
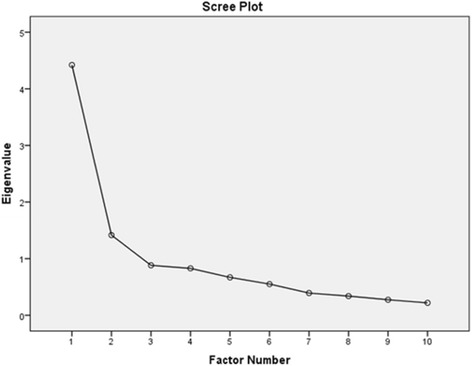
Scree plot of V-PSS-10 in factor analysis with extraction method: Principle Axis Factoring; rotation method: Promax with Kaiser Normalisation

All diagnosis tests indicated sample adequacy for proceeding with EFA. To be specific, the KMO measure was found to be 0.849 with Barlett’s test of sphericity significant with (*χ* ^2^/df) = 1995.960/45, *p* < .01. With regards to the dimensionality of the V-PSS-10, the scree plot showed that the curve levelled off after the first two components, with eigenvalues of 4.422, and 1.416, respectively, accounting for 44.223 and 14.164% of variance. These supported the two-factor model (See Fig. 1). The two factors had a significant correlation (0.56, *p* < .001), and item loadings exceeded .50 (see Table 1 for further details).

**Table 1:** Items and the standardised factor loadings based on EFA of the Vietnamese version of the Perceived Stress Scale 10 items (V-PSS-10) (*n* = 440)

		1	2
a.	…been upset because of something that happened unexpectedly?	.660	.041
b.	…felt that you were unable to control the important things in your life?	.581	-.242
c.	…felt nervous and “stressed”?	.645	-.010
d.	…felt confident about your ability to handle your personal problems?	.054	.742
e.	…felt that things were going your way?	.561	.375
f.	…found that you could not cope with all the things that you had to do?	.825	-.145
g.	…been able to control irritations in your life?	-.361	.671
h.	…felt that you were on top of things?	.710	.242
i.	…been angered because of things that were outside of your control?	.553	-.180
j.	…felt difficulties were piling up so high that you could not overcome them?	.781	-.066

Extraction method: Principal axis factoring method: Promax with kaiser normalization

Among older women in Vietnam, the perceived stress scores ranged from 0 to 34 (median =6.0); the general sleep disturbance scores ranged from 0 to 91 (median =29.0); the CES-D score for depression symptoms ranged from 0 to 52 (median =5.0); and the physical and mental health scores ranged from 10.37 to 61.81 (median =34.29) and 17.13 to 69.77 (median =59.41) respectively. The perceived stress scores were positively correlated with general sleep disturbance scores (*ρ* = .12, *p* < .05), depression symptom scores (*ρ* = .60, *p* < .01), and negatively correlated with mental (*ρ* = −.46, *p* < .01), and physical health (*ρ* = −.19, *p* < .01) scores.

### Internal consistency and test-retest reliability

The Cronbach’s alpha value for the V- PSS-10 among 473 participants was .80. The questionnaire was administered again a month later (Time 2) on a sample of 28 participants of the 473 original participants. Overall, participants in the retest were aged 60 – 84 years (median = 69.0). The majority had completed primary school or less (64.3%, *n* = 18), were not currently employed (75.0%, *n* = 21), had a moderate income or less (92.9%, *n* = 26), and lived with their partners (53.6%, *n* = 15). The test-retest correlation coefficient was 0.43 at the one month follow up.

## Discussion

The PSS-10 is one of the most widely used generic measures of stress, having been translated into more than 20 languages and used in different populations [14]. However, this is the first study to translate rigorously PSS-10 into Vietnamese, and to examine the translation equivalence, and to evaluate psychometric properties of the V-PSS-10 in older women. The V-PSS-10 is available on request from the first author. Overall, findings from this study suggest that the V-PSS-10 attained very good translation equivalence and face validity. In addition, the exploratory factor analysis on data of 473 older women showed that the V-PSS-10 has a two-factor structure. All items had loadings exceeding .50 on one of the two factors identified in this current study. This finding indicated that all items contributed significantly to measuring the perceived stress concept among older women.

However, previous studies indicated that Factor 1 included 6 items about general distress; Factor 2 included 4 items about ability to control. Inconsistently, the current study found that Factor 1 factor included 8 items, and Factor 2 included 2 items. Two items (“felt that things were going your way” and “felt that you were on top of things”), which were loaded on Factor 2 in previous studies, were in Factor 1 in the current study. This difference might be related to the socio-economic background of the study participants and the cultural differences in the interpretation of these two items’content. As seen in the descriptive statistics, the majority of Vietnamese older women had low education, and income. In addition, currently, there is very limited support from the Government for older people in Vietnam [37]. Thus, many of Vietnamese older women may have lived dependently on their families. Furthermore, generally Vietnamese people believe in Buddhism principles [38], which indicate it is your fortunate destiny to have “things going your way” or “being on top of things”. For these people, these two items may be about how things happened to them but not about their ability to control them. In addition, traditionally, Vietnamese women were taught “to obey their fathers when they are young, obey their husbands when they get married, obey their son when their husbands pass away” [39]. This tradition of Vietnamese women may have contributed to Vietnamese women avoiding confrontation, or to be less involved in fighting for the best options. These factors therefore could explain why these two items dropped from the Factor 2. However, the possible explanations have not been investigated in previous studies. It would be useful to test if there are significant differences in distress, and coping behaviours across gender, age groups, education levels, incomes and religions in future studies.

Apart from the difference in the factor loading, the current study finding is consistent with previous findings, indicating a significant correlation between factors (.56). Previous studies also reported a strong correlation between two factors among Chinese policemen (r = −.47) [40] and among Chinese cardiac patients (r = −.57) [41]. This strong correlation suggests that there might be an overlap among the items in these two factors. In addition, only two items for Factor 2 appear not strong enough to use as a subscale [42]. A further analysis of internal consistency reliability of these two items found a Cronbach’s alpha of .55. This value indicated a low reliability of a subscale having only these two items. Therefore, caution is necessary with using two dimensions of the construct as subscales individually. This finding supports Cohen’s (the original developer of the instrument) recommendation to use all 10 items to measure perceived stress rather than the statistically indicated sub-scales individually [13].

In regards to the correlates, in line with previous studies, the current study found small correlations between perceived stress and physical health [43], and sleep disturbance [44], and large correlations between perceived stress and depression [45–47]. While the magnitude of the correlation between perceived stress and mental health was large in a previous study [43], it was moderate in the current study. These findings suggest that the influences of perceived stress on sleep disturbance and physical health were weak among Vietnamese older women. However, Vietnamese older women, who had higher levels of perceived stress, were also much more likely to experience a higher level of depression and decreased mental health.

Finally, the internal consistency reliability value of the V-PSS-10 (alpha coefficients = .80), which was found in this study, is comparable with other studies’ findings [14] and passed the desirable value [36]. However, the test-retest reliability value of the V-PSS-10, which was found in this study, was lower than previous studies’ findings (.43 vs > .70) [14]. Previous studies measured test-retest reliability of PSS-10 at only 1 and 2 weeks’ interval. Consequently, the participants may have still remembered what they had answered in the first round. In contrast, the current study measured the test-retest reliability a month time apart. Hence, the participants’ experience of stress may have changed in a month. Therefore, the length of time for test-retest might be the reason why the test-retest reliability in this study was lower compared to previous studies’ findings.

### Study limitations

Several limitations should be noted. First, the scale was tested for the psychometric properties in Vietnamese older women and therefore the generalisations of the current study may be applicable only for similar population groups. As a qualitative step was not performed to evaluate the content’s relevance of the scale to Vietnamese culture, relevance of the content of the instrument to measure the perceived stress concept among older women in Vietnam is unclear. Also, as psychometric testing has been done solely on older women, the study findings could not be compared across genders, and age groups. Further, the current study used self-reported measures, recall and reporting bias may occur. In addition, data stemmed from a cross-sectional design and therefore prospective predictive validity of the PSS-10 could not be performed.

## Conclusion

The current study provides robust evidence that the V-PSS-10 has acceptable validity and reliability levels for older women and can be used to measure stress in future research and practice among Vietnamese people, who have similar characteristics with the study population. The use of the V-PSS-10 provides a valuable opportunity to explore perceived stress and its correlates in different Vietnamese populations. Moreover, as the PSS-10 now has been translated and validated in more than 20 languages, use of V-PSS-10 will provide additional opportunities for cross-cultural comparison studies between Vietnamese and people from other cultures. However, future studies would be beneficial to further endorse the validity and reliability of the V-PSS-10.

## Abbreviations

CES-D: The Centre of Epidemiologic Studies Depression Scale
EFA: Exploratory factor analysis
GSDS: General sleep disturbance scale
KMO: Kaiser-Mayer-Olkin
MCS: Mental health component scores
PCS: Physical health component scores
PSS-10: Perceived stress scale 10 items
SF12: The short-form 12 items
SPSS: Statistical package for social sciences
V-PSS-10: The vietnamese version of the perceived stress scale 10 items
